# Genetic susceptibility to infectious disease in East African Shorthorn Zebu: a genome-wide analysis of the effect of heterozygosity and exotic introgression

**DOI:** 10.1186/1471-2148-13-246

**Published:** 2013-11-09

**Authors:** Gemma GR Murray, Mark EJ Woolhouse, Miika Tapio, Mary N Mbole-Kariuki, Tad S Sonstegard, Samuel M Thumbi, Amy E Jennings, Ilana Conradie van Wyk, Margo Chase-Topping, Henry Kiara, Phil Toye, Koos Coetzer, Barend M deC Bronsvoort, Olivier Hanotte

**Affiliations:** 1 Institute of Evolutionary Biology, and Centre for Immunity, Infection and Evolution, University of Edinburgh, Edinburgh EH9 3JT, UK; 2 Department of Genetics, University of Cambridge, Cambridge, Downing Street, Cambridge CB2 3EH, UK; 3 MTT Agrifood Research Finland, Biotechnology and Food Research, Jokioinen FI-31600, Finland; 4 School of Life Sciences, University of Nottingham, University Park, Nottingham NG7 2RD, UK; 5 United States Department of Agriculture, Agricultural Research Service, Bovine Functional Genomics Laboratory, 10300 Baltimore Avenue, Beltsville, MD 20705, USA; 6 Roslin Institute, Easter Bush, University of Edinburgh, Roslin, Midlothian, EH25 9RG, Edinburgh, UK; 7 Department of Veterinary Tropical Diseases, Faculty of Veterinary Science, University of Pretoria, Private bag X04, Onderstepoort, South Africa; 8 International Livestock Research, P.O. Box 30709, Nairobi 00100, Kenya; 9 Current address: Paul G Allen School for Global Animal Health, Washington State University, Pullman, WA 99164-7079, USA

**Keywords:** Inbreeding depression, Heterozygosity, Fitness, Infectious disease, Exotic introgression, Local adaptation, Cattle

## Abstract

**Background:**

Positive multi-locus heterozygosity-fitness correlations have been observed in a number of natural populations. They have been explained by the correlation between heterozygosity and inbreeding, and the negative effect of inbreeding on fitness (inbreeding depression). Exotic introgression in a locally adapted population has also been found to reduce fitness (outbreeding depression) through the breaking-up of co-adapted genes, or the introduction of non-locally adapted gene variants.

In this study we examined the inter-relationships between genome-wide heterozygosity, introgression, and death or illness as a result of infectious disease in a sample of calves from an indigenous population of East African Shorthorn Zebu (crossbred *Bos taurus* x *Bos indicus*) in western Kenya. These calves were observed from birth to one year of age as part of the Infectious Disease in East African Livestock (IDEAL) project. Some of the calves were found to be genetic hybrids, resulting from the recent introgression of European cattle breed(s) into the indigenous population. European cattle are known to be less well adapted to the infectious diseases present in East Africa. If death and illness as a result of infectious disease have a genetic basis within the population, we would expect both a negative association of these outcomes with introgression and a positive association with heterozygosity.

**Results:**

In this indigenous livestock population we observed negative associations between heterozygosity and both death and illness as a result of infectious disease and a positive association between European taurine introgression and episodes of clinical illness.

**Conclusion:**

We observe the effects of both inbreeding and outbreeding depression in the East African Shorthorn Zebu, and therefore find evidence of a genetic component to vulnerability to infectious disease. These results indicate that the significant burden of infectious disease in this population could, in principle, be reduced by altered breeding practices.

## Background

Positive multi-locus heterozygosity-fitness correlations have been observed in a number of natural populations [1,2]. Genome-wide heterozygosity is correlated with inbreeding within a population [3] and heterozygosity-fitness correlations have therefore been associated with inbreeding depression [4]. Inbreeding may be detrimental to fitness if: (i) fitness related traits are over-dominant, such that the heterozygous genotype results in greater fitness than either homozygote, or (ii) there are recessive alleles present in the population that result in lower fitness [5]. The extent to which the heterozygosity of genetic markers reflects genome-wide heterozygosity and inbreeding is a contentious issue, and heterozygosity-fitness correlations may result from a few loci of major effect in linkage disequilibrium with the genetic markers rather than the effect of inbreeding across the genome [2,3,6,7]. However, when large numbers of markers are used, estimates of genome-wide heterozygosity are greatly improved, are more likely to correlate with inbreeding, and are less likely to correlate with the fitness effects of a few loci [2,3].

Inbreeding has been observed to have a negative effect on fitness-related traits in many populations [8], e.g. [9,10], including some European cattle populations [11,12]. In a number of natural populations susceptibility to infectious disease has been found to be associated with heterozygosity e.g. [13-15] and inbreeding e.g. [16]. Infectious disease susceptibility in humans has been found to have a strong genetic component in numerous studies reviewed in [17], with many genes and genetic pathways associated with susceptibility to different diseases. The major histocompatibility complex (MHC) is considered to play a significant role in determining susceptibility to various pathogens [18], and MHC-targeted breeding programmes have been suggested for captive populations [19,20]. In one natural population it was found that the effect of a single locus linked to the MHC exceeded the effect of genome-wide heterozygosity on fitness [21].

Exotic introgression in a locally adapted population may also reduce fitness. A number of studies have observed a negative effect of introgression on fitness (outbreeding depression) [22,23]. These may result from either (i) the break-up of co-adapted epistatic interaction between genes, or (ii) the introduction of non-locally adapted alleles [24].

In this study, we tested for associations of heterozygosity and exotic European taurine breed introgression with death and illness due to infectious disease during the first year of life, in a population of East African shorthorn zebu (EASZ) cattle (crossbred *Bos taurus* x *Bos indicus*) from western Kenya. More than 500 calves were followed for the first year of their life as part of the Infectious Disease in East African Livestock (IDEAL) project [25]. The calves were closely monitored throughout; deaths and episodes of clinical illness were reported and followed up by trained veterinary staff. During the study, more than 15% of the calves died, with the majority of deaths due to infectious diseases, most commonly East Coast Fever, helminth infections, heartwater and trypanosomiasis [26]. Given the heavy infectious disease burden on these cattle coupled with minimal disease control or treatment, an ability to survive infection is likely to be significantly associated with fitness. Furthermore, more than half the surviving calves were reported to have at least one episode of clinical illness during the study period, and almost all these episodes were attributed to infectious disease (see Methods). We propose that clinical illness is also indicative of a vulnerability to infectious disease that could affect fitness in this environment.

All calves in the IDEAL population were genotyped using the 50K Illumina® BovineSNP50 beadchip v. 1 (55,777 SNPs before quality control). These SNPs are relatively evenly distributed across the genome, with an average of 1,895 on each autosome (ranging from 1,009 to 3,553) and 1,362 on the X chromosome (102 remain unassigned).

The study population was found to be partly introgressed by one or possibly more European taurine breeds [27,28] (determined from analysis of genome-wide SNP data using STRUCTURE [29]). This reflects the effects of a substantial breed improvement programme, involving the crossing of local cattle with European breeds, that took place in the mid-1990s with intermittent cross-breeding thereafter. As a result, approximately 20% of the calves show levels of introgression consistent with crossing with European breeds ≤ 5 generations ago (fraction European taurine > 2^-6^) [27].

We would also expect some inbreeding to occur in this population, since mating is largely unmanaged, there is a relatively low number of breeding bulls, and cattle are generally only transported over short distances (only a small fraction of cattle are traded and these over distances typically just 20-30 km). Also, historically, this region has experienced major rinderpest epidemics, which have reduced the effective population size of the EASZ [28]. In this study we calculate the genome-wide heterozygosity for the calves in the IDEAL population using the same SNP data set as was used to calculate exotic introgression [28].

In this study we aim to determine whether inbreeding (as estimated by SNP heterozygosity) and outbreeding (as estimated by the estimated proportion of genetic markers that descend from a European taurine breed) affect the likelihood of death or illness due to infectious disease and therefore whether there is evidence of a genetic basis to vulnerability to infectious disease in EASZ. We use results from earlier studies that have determined the degree of European taurine introgression [28] and describe and analyse the causes of instances of death and illness for all the calves in the IDEAL project [25,26]. Through analysis of these data we find associations of both heterozygosity and European taurine introgression with vulnerability to infectious disease during the first year of life in this population of EASZ.

## Results

The sample size available for analysis was 518 calves (see Methods for inclusion/exclusion criteria), of which 68 (13%) died of infection-related disease within one year of birth (see Methods for more details). Of the survivors, episodes of clinical illness (almost always related to infections) were reported in 243 (54%) (Table 1, Additional file 1: Table S1).

**Table 1:** Numbers of calves that fall into the different categories used for the statistical analysis of the effects of heterozygosity and introgression on likelihood of death and clinical illness

	**Low heterozygosity**		**High heterozygosity**	
	**Introgressed**	**Non-introgressed**	**Introgressed**	**Non-introgressed**
**Died**	2	11	11	44
**Survived with clinical episode**	6	13	64	160
**Survived without clinical episode**	1	4	29	173

Cut-offs for high/low heterozygosity and introgressed/non-introgressed are defined in Methods.

**Figure 1:**
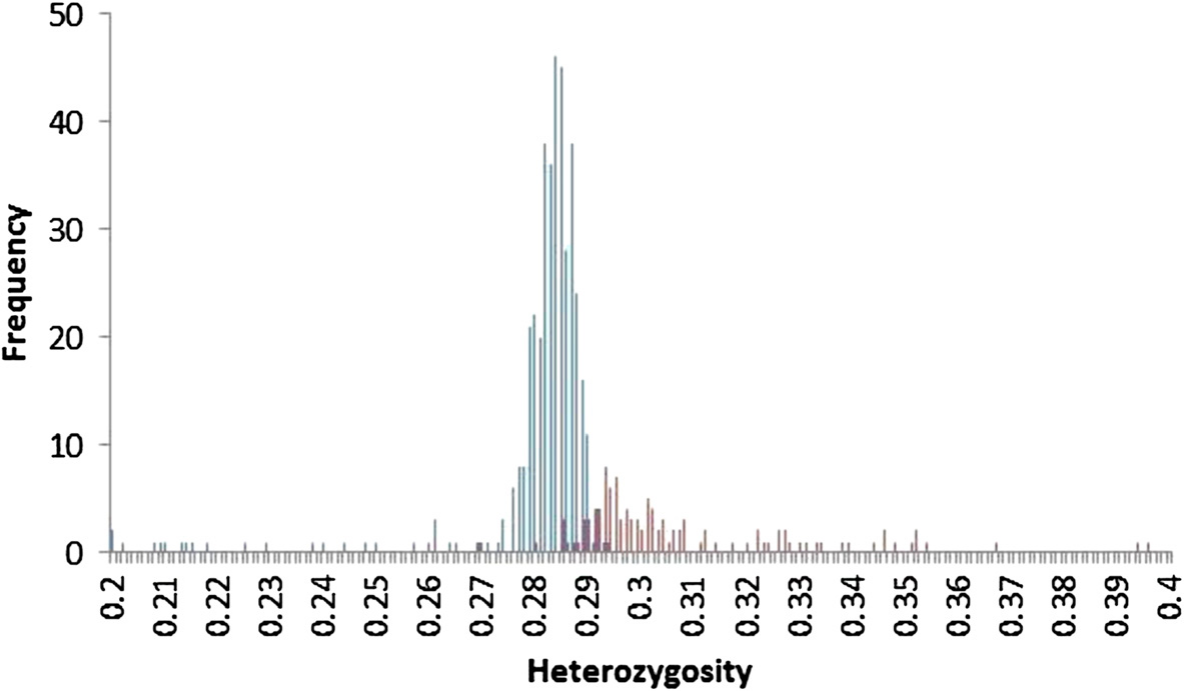
Histogram of frequency of heterozygosity values for European taurine introgressed (red) and non-introgressed (blue) East African Shorthorn Zebu calves.

The distributions of observed heterozygosity values for non-introgressed (defined as < 2^-6^ European taurine) and introgressed calves (> 2^-6^ European taurine) are shown in Figure 1. The mean heterozygosity of non-introgressed calves is 0.28 (with a standard deviation (SD) of 0.01). This is not an unusual level of heterozygosity for a cattle population. Holstein-Friesians (European taurine) have been found to have average heterozygosity of 0.33 (SD = 0.01), Jersey (European taurine) have an average heterozygosity of 0.25 (SD = 0.03), N’dama (African taurine) have an average heterozygosity of 0.17 (SD = 0.03) and Ethiopian Sheko (admixed African taurine and Asian zebu) have an average heterozygosity of 0.26 (SD = 0.0003) [27].

In order to test whether or not our estimates of heterozygosity are likely to reflect true genome-wide heterozygosity, we randomly sampled our SNPs without replacement 100 times and determined the degree of correlation in the heterozygosity estimates for calves based on one half of the markers and the estimates based on the other half [2,6]. We found a consistently strong correlation between the estimates (mean Pearson’s r = 0.98, SD = 0.0012, range = 0.977-0.982). Since some SNPs may be in linkage disequilibrium with one another, we repeated the analysis with two smaller subsets (each representing a different random sample, without replacement, of one 10^th^ of the total SNPs). Even with this much smaller set of markers, a strong correlation between the estimates was found (mean Pearson’s r = 0.91, SD = 0.0053, range = 0.89-0.92).

The non-introgressed calves show a skewed distribution of heterozygosity values, with the majority (93%) contained within a near-symmetrical distribution ± 0.010 from a mode of 0.284 (Figure 1). However, there is a long tail of 28 calves with lower values. We categorise calves with values < 0.274 as “low” heterozygosity, assumed to reflect a higher degree of inbreeding than the population norm. Introgressed calves show a different distribution of heterozygosity values that are generally higher than those for non-introgressed calves (Figure 1) (Mann Whitney U test, *P* < 0.001).

**Figure 2:**
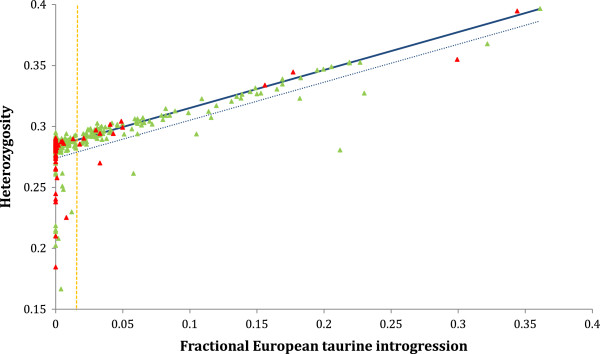
**Plot of heterozygosity against proportion European introgression for all calves in our analysis.** Calves that died are highlighted (red symbols). The best fit relationship between heterozygosity (H) and introgression (I) for non-inbred calves is shown (thick line: regression equation H = 0.284 (95% confidence intervals ±0.0003) + I*0.312 (±0.006), R^2^ = 0.972). The cut-off to define ‘low’ heterozygosity calves varies with introgression, since the expected or mean heterozygosity varies with introgression. Calves that are more that 0.01 below the expected heterozygosity (blue dashed line) for their amount of introgression are defined as ‘low’ heterozygosity (see Results and Methods). The orange dashed line indicates the cut-off for introgressed calves (2^-6^%).

The relationship between heterozygosity and introgression is shown in Figure 2. As expected, there is a clear increase in heterozygosity with increasing introgression. However, in addition to the 28 non-introgressed calves with low heterozygosity, there are several introgressed calves with heterozygosity values that appear low relative to the amount of introgression. In order to investigate the effect of variation in heterozygosity on the fitness of introgressed calves independently of the effect of introgression we define a group of calves with low heterozygosity relative to their degree of introgression. We define this group by finding the best fit linear regression and iteratively excluding calves with heterozygosity values > 0.01 below their expected value. This procedure results in the fitted regression line shown in Figure 2 (with the -0.01 threshold also indicated). Using this criterion, 9 of the 113 introgressed calves are categorised as low heterozygosity. Two of these 9 died and 6 others experienced at least one clinical episode (see Table 1).

We compared both calves that died and those that survived but experienced any episode of clinical illness to a reference category of healthy calves (those that survived and had no reported episode of clinical illness). We considered two categorical predictors of these outcomes, low heterozygosity and being introgressed, using a multinomial logistic regression (described in Methods; results in Table 2). We used these categorical predictors, rather than analysing the estimates directly, due to the non-normality of the data (the distribution of values of both heterozygosity and introgression are highly skewed) and to allow us to robustly account for the relationship between heterozygosity and introgression (Figures 1 and 2, Additional file 1: Table S1).

**Table 2:** Summary of results of a multinomial logistic regression of the effects of genome-wide heterozygosity and introgression (as two level factors) on the likelihood of death and the likelihood of clinical illness

**Phenotype**	**Dead calves (n = 68)**			**Ill calves (n = 243)**		
**Factors**	**Estimate (SE)**	**OR (95% CI)**	***P*-value**	**Estimate (SE)**	**OR (95% CI)**	***P*-value**
**Low heterozygosity**	1.13 (0.27)	9.5 (3.5-27.9)	**< 0.001**	0.61 (0.26)	3.4 (1.2-9.3)	**0.018**
**Introgressed**	0.16 (0.19)	1.4 (0.7-2.9)	0.39	0.43 (0.12)	2.4 (1.5-3.8)	**< 0.001**

Cut-offs for high/low heterozygosity and introgressed/non-introgressed are defined in Methods. Healthy calves were the reference category. CI = confidence interval. SE = standard error. OR = odds ratio. *P*-values in bold type are statistically significant.

We first consider the association between predictors and calf death due to infectious disease. For non-introgressed calves (fraction European taurine < 2^-6^) only there is strong evidence of a relationship between death and low heterozygosity: odds ratio (OR) = 10.8 (95% CIs 3.0-48.1); *P* < 0.001 (Table 3). For all calves, this relationship remains when both low heterozygosity and introgression are included as predictors: OR = 9.5 (3.5-27.9); *P* < 0.001 (Table 2). There is no evidence of an interaction between low heterozygosity and introgression (*P* > 0.05) and there is no evidence of an association between death and degree of introgression (OR = 1.4 [0.7-2.9]; *P* = 0.39) (Table 2). No significant differences were found in the nature of infectious disease-related deaths among the low heterozygosity calves. These results are robust to variation in the cut-off for introgressed calves (see Methods).

**Table 3:** Summary of results of Fisher’s exact tests of the effects of low genome-wide heterozygosity (as a two-level factor) on the likelihood of death and the likelihood of clinical illness for the sub-populations of non-introgressed and introgressed calves

**Phenotypes**	**Dead vs healthy calves**			**Ill vs healthy calves**		
**Subpopulation**	**OR**	**95% CI**	***P*-value**	**OR**	**95% CI**	***P*-value**
**Non-introgressed**	10.8	3.0-48.1	**< 0.001**	3.5	1.05-15.1	**0.026**
**Introgressed**	5.3	0.24-321	0.213	2.72	0.31-129	0.671

Cut-offs for high/low heterozygosity and introgressed/non-introgressed are defined in Methods. OR = odds ratio. CI = confidence interval. *P*-values in bold type are statistically significant.

Clinical illness is a less well defined phenotype than death, since it includes a wide range of disease signs and it cannot be unequivocally determined whether or not an instance of clinical illness was the result of infectious disease or not (although it is likely that the overwhelming majority were). Despite this, clinical illness and death are clearly related outcomes. Moreover, because many more calves experienced clinical illness during their first year than died, this outcome may provide increased power to detect small effects. For non-introgressed calves there is evidence of a relationship between illness and low heterozygosity: OR = 3.5 (95% CIs 1.05-15.1); *P* = 0.026 (Table 3). For all calves, this relationship remains when both low heterozygosity and introgression are included as predictors: OR = 3.4 [1.3-9.3]; *P* = 0.018 (Table 2). However, for this phenotype there is also strong evidence of an association with introgression, with introgressed calves being more likely to experience illness (OR = 2.4 [1.5-3.8]; *P* < 0.001). Again there is no evidence of any interaction between low heterozygosity and introgression (*P* > 0.05). These results are robust to variation in the cut-off for introgressed calves (see Methods).

## Discussion

Our results indicate that there is a genetic basis to vulnerability to infectious disease in East African Shorthorn Zebu. We find a positive association between low heterozygosity and both death and illness. Since we use a reasonable proxy for genome-wide heterozygosity (40,457 SNPs across all autosomes), and the estimates of genome-wide heterozygosity are highly correlated for different subsets of SNPs, we consider it likely that our results are indicative of the presence of inbreeding depression in the study population, rather than being a consequence of localised associations between fitness and loci of large effect [6,7,17].

Our results indicate the presence of fitness-related genotypes that are either over-dominant or have recessive deleterious alleles in the population. Although the proportion of calves that we classified as “low” heterozygosity was small (approximately 7%), the effect on infectious disease-related mortality was large (OR ~ 9) and there was also an effect on clinical illness (OR ~ 3). Our results give population attributable fractions (for the non-introgressed population) due to low heterozygosity of 14% of mortality and 4% of clinical illness [30]. In principle, therefore, cases of illness and deaths could be reduced by better management practices (e.g. bull rotation).

We found evidence that European taurine introgression affected health as it was a significant predictor of clinical illness (Table 2). As there were only 13 deaths among those with fractional introgression greater than 2^-6^ we had very little power to detect any effect on death alone. Nonetheless, our results for these two phenotypes are not inconsistent, as the confidence intervals for the odds ratios overlap (see Table 2). We suggest that clinical illness is a marker for vulnerability in this population and consequently that European taurine introgressed calves are more vulnerable in this sense.

We could find no non-genetic confounding factors that could be driving the observed associations between introgression and either death or illness. The introgressed calves (even those with high introgression) could not be distinguished from non-introgressed calves by the field team [25]; exotic introgression was only discovered upon genetic analysis of the calves. And, although the introgressed calves were not found uniformly distributed among the 20 sub-locations of the study [28], there is no evidence of geographic variation in the rate of mortality or of clinical illness.

Since the EASZ ancestors of the study population have been present in East Africa for many generations [28], it might be expected that they may have in some way adapted to the specific challenges posed by that environment, in particular to the infectious diseases present in that region. Infectious disease was the primary cause of first year mortality and illness in this population, and thus the ability to withstand infection is likely to be of significant selective advantage. On the other hand, the introgressed animals descend, in part, from European cattle, which have not had the same history of selective pressures. It has been found that European breeds are more susceptible than local breeds to some infectious diseases that are prevalent in Africa, such as East Coast Fever [31,32], bovine tuberculosis [33,34], and trypanosomiasis [35,36]. Thus, the European breeds are, in this important respect, less well adapted to the East African environment, and introgressed animals, while more heterozygous due to outbreeding, are also less locally adapted. While increased heterozygosity is likely to be beneficial if fitness related traits are over-dominant or there are recessive deleterious alleles in the population, the breaking up of or loss of locally adapted genotypes is likely to be detrimental to fitness. Both these competing forces appear to be at work in this population of East African Shorthorn Zebu calves in western Kenya.

## Conclusions

In conclusion, in this indigenous cattle population from the tropics we find strong associations between genome-wide heterozygosity and both death and clinical illness due to infectious diseases. There is evidence that introgression of European taurine breeds also increases vulnerability to infectious diseases. We therefore believe that we have observed the effect of both inbreeding and outbreeding depression, and have found evidence of a genetic component to vulnerability to infectious disease and of adaptation in the East African Shorthorned Zebu population in western Kenya. This implies that the significant burden of infectious disease in this population could, in principle, be reduced by better breeding practices.

## Methods

### Data set

The IDEAL study population consisted of 548 calves, which were monitored closely throughout their first year of life [25]. Three of these calves were excluded from this analysis because they received treatment for disease. The calves were sampled from a region in western Kenya (of approximately 45 × 90 km) (covering some of the Busia, Teso, Siaya, Butere/Mumias and Bungoma districts). This region traversed four different types of agro-ecological zones. The region was divided into sub-locations of approximately 10 × 10 km, of which 20 were randomly selected (within each agro-ecological zone) for study [25]. Small-scale cattle farming is common in this region and the sub-locations contain approximately 80 to 90 households, of which approximately 60% own cattle (generally ranging from 1-10 cattle per household). New born calves were recruited into the study from these 20 sub-locations over a period of 2 years (2007-2009). Calves that showed visible signs of exotic breed introgression were excluded, and only calves from small farms, where the cattle spent at least part of their time outside, exposed to infectious disease, were recruited [25].

### Genetic data

All calves in the IDEAL population were genotyped using the 50K Illumina® BovineSNP50 beadchip v. 1 including 55,777 SNPs before quality control. Standard quality control procedures were applied to the data prior to analysis reviewed in [37]. This was implemented using GenABEL. A minor allele frequency for SNPs cut-off of 1% was applied and a SNP call-rate cut-off of 90%. A cut-off of call rate for an animal of 90% was applied. And an identity by state (IBS) threshold of 90% and a cut-off odds of 1000-1 was applied to decide whether a calf should be excluded based on sex/X-linked marker data inconsistency (which both eliminate errors due to sample mis-identification). These quality control thresholds left a total of 40,457 autosomal SNPs for subsequent analysis. A Hardy-Weinberg equilibrium (HWE) cut-off was not applied, since a divergence from HWE can result from factors other than genotyping error (in this case introgression).

As a result of quality control, 9 calves and 13,856 of the 54,313 initial autosomal SNPs had to be excluded from the 50K SNP chip data set due to unsuccessful genotyping or mis-identification of individuals. This reduced the genotyping data set for this study to 536 calves and 40,457 autosomal SNPs.

### Fitness phenotype – death

The best proxy for fitness in this study is survival to first year. Eighty-eight of the original 548 calves died during the study, and all but nine of these were subject to a detailed post-mortem examination [25,26]. In most cases the cause of death was found to be infectious disease. These results are described in detail elsewhere [25,26], but the most frequent causes of death were East Coast Fever (caused by the tick-borne protozoan *Theileria parva*), heartwater (caused by the tick-borne rickettsia *Ehrlichia ruminantium*) and helminthosis (especially associated with the nematode *Haemonchus placei*). Of the 88 deaths, 9 were found to have causes unlikely to be associated with any heritable fitness, such as trauma and starvation, and 10 had an undetermined cause of death. These calves were excluded from the analysis, so as to make the phenotype more likely to have a consistent (although likely complex) genetic basis. This resulted in a data set, after quality control, of 68 calves that died due to infectious disease, and 450 calves that survived their first year (Table 1, Additional file 1: Table S1). Follow-up studies indicated that the death rate over the next 1-2 years was much lower than during the first year.

### Fitness phenotype – clinical illness

All calves were monitored for signs of clinical illness throughout the study. Episodes of clinical illness were reported by the calf’s owner, visiting animal health workers or the project staff and were subject to detailed investigation using a standardised protocol and, where necessary, followed up by the project’s veterinary staff [25]. Of the surviving calves in the study, 243 experienced at least one reported episode of clinical illness. Clinical signs ranged from mild to severe. Treatment was provided if the calf was in distress and its keeper agreed; treated calves (n = 3) were subsequently excluded from the study. In a few instances a severely ill calf was euthanized; this was regarded as equivalent to a death. Almost all episodes of clinical illness could be attributed to infections, however causal links are difficult to definitively establish and so episodes cannot be unequivocally divided into those that were caused by infectious disease and those that were not. For this reason all episodes of clinical illness were used to distinguish between healthy and unhealthy calves (where this difference in health can generally be attributed to variation in susceptibility to infectious disease). This division results in 207 calves that neither died nor experienced any reported episodes of clinical illness up to one year old.

### European taurine introgression

Using a cut-off of fraction 2^-6^ introgression we identified 113 introgressed calves, with a maximum of 36.1% European taurine introgression. The introgressed calves were not found uniformly distributed among the 20 sub-locations of the study: they are found mainly in 12 sub-locations in the northern and central regions of the study area [28]. The most likely origin of this European taurine introgression in the study area is a number of recent and continuing breed improvement programmes that use exotic animals and semen, and cattle markets in the study area continue to sell crossbred cattle [28]. The most active programme took place in the mid 1990s, equivalent to approximately 5 EASZ generations before our study took place. This is reflected in our cut-off of fraction 2^-6^ introgression, corresponding to the expected level of introgression after 5 generations of back-crossing with indigenous cattle since the F1 generation. However, to confirm the robustness to the precise value of the cut-off, we repeated all analyses using alternative cut-off values but obtained very similar results.

### Heterozygosity

Individual mean genome-wide heterozygosity was computed as the proportion of all autosomal genotyped loci that passed quality control (40,457 SNPs) that were heterozygous. This was implemented using GenABEL [38]. To test for the whether this is likely to be a good estimate of genome-wide heterozygosity, we randomly sampled SNPs without replacement 100 times and calculated the Spearman correlation coefficient of the heterozygosity estimates for calves based on one half of the markers and the estimates based on the other half [2,6]. Since SNPs may in linkage disequilibrium with one another we repeated the analysis comparing smaller subsets.

### Statistical methods

The comparisons between healthy calves (n = 217) and those that died as a result of infectious disease (n = 68) or survived but experienced clinical illness (n = 243) was investigated using a 3-level multinomial logistic regression analysis using Proc Logistic SAS version 9.3 (SAS Institute Inc., Cary, NC). Both heterozygosity and introgression were included as predictors and were categorised for the purposes of analysis. Using a cut-off of fraction 2^-6^ introgression we identified 113 introgressed calves. We repeated all analyses using alternative cut-offs of 2^-7^, 2^-5^ and 2^-4^ (6, 4 and 3 generations of crossing respectively). We categorised non-introgressed calves with heterozygosity < 0.274 (more than 0.10 below the mean for this group) as low heterozygosity, assumed to reflect a higher degree of inbreeding than the population norm (28 calves). We defined low heterozygosity calves in the introgressed group by finding the best fit linear regression of heterozygosity against introgression and iteratively excluding calves with heterozygosity values > 0.01 below their expected value (resulting in 9 of the 113 introgressed calves being categorised as low heterozygosity).

We conducted confirmatory univariate analyses using Fisher’s exact test (StatXact version 8, Cytel Software Corp, Cambridge, MA, USA) for the non-introgressed and introgressed categories, allowing us to investigate the effect of heterozygosity for these separately.

## Availability of supporting data

Genotyping data can be found at Dryad (http://datadryad.org/resource/doi:10.5061/dryad.bc598).

## Competing interests

The authors declared that they have no competing interests.

## Authors’ contributions

MW, OH, HK, PT, KC and MB designed the IDEAL project. GM, MW and OH drafted the manuscript. GM calculated estimates of heterozygosity. MM and MT calculated estimates of introgression. TS completed the SNP genotyping and SNP marker scoring. MCT, MW and GM did the statistical analysis. ST, AJ and IC did fieldwork for the IDEAL project, performed autopsies and diagnostic tests. All authors read and approved the final manuscript.

## Supplementary Material

Additional file 1: Table S1 Table describing heterozygosity (Het), proportion European taurine (%ET), whether they died during the study (and whether this is known to have been the result of an infectious disease) and whether they experienced one or more clinical episodes, for all of the calves included in this analysis. Click here for file
