# Supplementary material for: Genetic susceptibility to infectious disease in East African Shorthorn Zebu: a genome-wide analysis of the effect of heterozygosity and exotic introgression
Source: BMC Evol Biol. 2013 Nov 9;13:246. doi: 10.1186/1471-2148-13-246 (PMC3828575; doi:10.1186/1471-2148-13-246)
Supplement: Additional file 1: Table S1 — Table describing heterozygosity (Het), proportion European taurine (%ET), whether they died during the study (and whether this is known to have been the result of an infectious disease) and whether they experienced one or more clinical episodes, for all of the calves included in this analysis. [file 1471-2148-13-246-S1.docx]

**Supplementary material**

**Supplementary table 1.** Table describing heterozygosity (Het), proportion European taurine (%ET), whether they died during the study (and whether this is known to have been the result of an infectious disease) and whether they experienced one or more clinical episodes, for all of the calves included in this analysis.

| **Calf ID** | **Het** | **%ET** | **Died during study** | **Clinical episode(s) during study** |
| --- | --- | --- | --- | --- |
| CA010110001 | 0.311 | 0.099 | No | Yes |
| CA010110002 | 0.297 | 0.03 | Yes | Yes |
| CA010110004 | 0.304 | 0.0701 | No | Yes |
| CA010110005 | 0.309 | 0.079 | No | Yes |
| CA010110006 | 0.305 | 0.08 | No | Yes |
| CA010110007 | 0.282 | 0.004 | No | No |
| CA010110008 | 0.331 | 0.1489 | No | Yes |
| CA010110009 | 0.294 | 0.027 | No | Yes |
| CA010110010 | 0.281 | 0 | No | Yes |
| CA010110012 | 0.29 | 0 | Yes | Yes |
| CA010110013 | 0.294 | 0.0352 | No | Yes |
| CA010110014 | 0.312 | 0.114 | No | Yes |
| CA010110015 | 0.214 | 0 | No | Yes |
| CA010110016 | 0.288 | 0.008 | No | Yes |
| CA010110017 | 0.327 | 0.153 | No | Yes |
| CA010110018 | 0.306 | 0.061 | No | Yes |
| CA010110019 | 0.355 | 0.2994 | Yes | No |
| CA010110020 | 0.304 | 0.049 | Yes | Yes |
| CA010110021 | 0.27 | 0.033 | Yes | Yes |
| CA010110022 | 0.281 | 0.212 | No | Yes |
| CA010110023 | 0.261 | 0.058 | No | Yes |
| CA010110024 | 0.323 | 0.1382 | No | Yes |
| CA010110025 | 0.314 | 0.082 | No | No |
| CA010110026 | 0.327 | 0.15 | No | Yes |
| CA010110027 | 0.299 | 0.05 | Yes | No |
| CA010110029 | 0.309 | 0.085 | No | No |
| CA010210031 | 0.296 | 0.034 | No | No |
| CA010210032 | 0.29 | 0.021 | Yes | Yes |
| CA010210033 | 0.285 | 0.023 | No | No |
| CA010210034 | 0.288 | 0.023 | No | No |
| CA010210035 | 0.282 | 0 | No | No |
| CA010210036 | 0.284 | 0 | Yes | Yes |
| CA010210037 | 0.289 | 0.007 | No | No |
| CA010210038 | 0.283 | 0 | No | No |
| CA010210039 | 0.281 | 0 | No | Yes |
| CA010210040 | 0.293 | 0.015 | No | Yes |
| CA010210041 | 0.283 | 0 | No | Yes |
| CA010210042 | 0.294 | 0.0206 | No | Yes |
| CA010210043 | 0.283 | 0.005 | No | No |
| CA010210044 | 0.282 | 0 | No | No |
| CA010210045 | 0.29 | 0.031 | No | Yes |
| CA010210047 | 0.293 | 0.023 | No | Yes |
| CA010210048 | 0.29 | 0.007 | No | Yes |
| CA010210049 | 0.295 | 0 | No | No |
| CA010210050 | 0.281 | 0 | No | No |
| CA010210051 | 0.284 | 0 | No | No |
| CA010210052 | 0.288 | 0 | No | No |
| CA010210053 | 0.285 | 0 | No | Yes |
| CA010210055 | 0.288 | 0.003 | No | Yes |
| CA010210056 | 0.284 | 0 | No | No |
| CA010210057 | 0.303 | 0.042 | No | No |
| CA010210058 | 0.28 | 0 | No | Yes |
| CA010310061 | 0.286 | 0 | No | Yes |
| CA010310062 | 0.328 | 0.145 | No | Yes |
| CA010310063 | 0.353 | 0.219 | No | No |
| CA010310064 | 0.294 | 0.033 | Yes | No |
| CA010310065 | 0.283 | 0 | No | Yes |
| CA010310066 | 0.299 | 0.031 | No | No |
| CA010310067 | 0.296 | 0.036 | No | Yes |
| CA010310068 | 0.346 | 0.195 | No | Yes |
| CA010310069 | 0.281 | 0 | Yes | No |
| CA010310070 | 0.21 | 0 | No | Yes |
| CA010310071 | 0.285 | 0.002 | No | Yes |
| CA010310072 | 0.281 | 0.0088 | No | Yes |
| CA010310073 | 0.288 | 0 | No | Yes |
| CA010310074 | 0.304 | 0.063 | No | Yes |
| CA010310075 | 0.368 | 0.3218 | No | No |
| CA010310076 | 0.283 | 0.001 | No | Yes |
| CA010310077 | 0.291 | 0 | No | No |
| CA010310078 | 0.281 | 0 | No | Yes |
| CA010310079 | 0.294 | 0.008 | No | Yes |
| CA010310080 | 0.334 | 0.156 | Yes | Yes |
| CA010310081 | 0.284 | 0 | No | Yes |
| CA010310082 | 0.275 | 0 | Yes | Yes |
| CA010310083 | 0.292 | 0.027 | No | Yes |
| CA010310084 | 0.282 | 0 | No | No |
| CA010310085 | 0.292 | 0.026 | No | Yes |
| CA010310086 | 0.294 | 0.105 | No | Yes |
| CA010310087 | 0.293 | 0.022 | No | Yes |
| CA010310088 | 0.286 | 0 | No | No |
| CA020410091 | 0.288 | 0.021 | No | No |
| CA020410092 | 0.284 | 0.01 | No | Yes |
| CA020410093 | 0.294 | 0.043 | Yes | No |
| CA020410094 | 0.285 | 0 | No | Yes |
| CA020410095 | 0.288 | 0.005 | No | Yes |
| CA020410096 | 0.285 | 0.003 | No | No |
| CA020410097 | 0.283 | 0 | No | Yes |
| CA020410098 | 0.282 | 0.002 | No | Yes |
| CA020410099 | 0.295 | 0.029 | No | Yes |
| CA020410100 | 0.335 | 0.1691 | No | No |
| CA020410101 | 0.28 | 0 | No | No |
| CA020410102 | 0.286 | 0 | No | No |
| CA020410103 | 0.286 | 0.0064 | No | Yes |
| CA020410105 | 0.278 | 0 | No | No |
| CA020410106 | 0.284 | 0.001 | No | Yes |
| CA020410107 | 0.294 | 0.061 | No | Yes |
| CA020410108 | 0.294 | 0.051 | No | Yes |
| CA020410109 | 0.297 | 0.025 | No | No |
| CA020410110 | 0.292 | 0.014 | No | Yes |
| CA020410111 | 0.301 | 0.064 | No | No |
| CA020410112 | 0.302 | 0.046 | No | Yes |
| CA020410113 | 0.29 | 0.038 | No | No |
| CA020410114 | 0.293 | 0.032 | No | No |
| CA020410116 | 0.284 | 0.001 | No | Yes |
| CA020410117 | 0.288 | 0 | No | No |
| CA020410118 | 0.248 | 0.0058 | No | No |
| CA020410119 | 0.293 | 0.029 | No | No |
| CA020510121 | 0.286 | 0.006 | No | Yes |
| CA020510122 | 0.296 | 0.042 | No | No |
| CA020510123 | 0.288 | 0.002 | No | No |
| CA020510124 | 0.289 | 0.007 | No | No |
| CA020510126 | 0.285 | 0 | No | No |
| CA020510127 | 0.28 | 0 | No | No |
| CA020510128 | 0.295 | 0.04 | No | No |
| CA020510129 | 0.283 | 0 | No | Yes |
| CA020510130 | 0.284 | 0 | No | No |
| CA020510131 | 0.284 | 0.005 | No | No |
| CA020510132 | 0.287 | 0 | No | No |
| CA020510133 | 0.289 | 0 | No | Yes |
| CA020510134 | 0.309 | 0.082 | No | No |
| CA020510135 | 0.288 | 0.001 | No | Yes |
| CA020510136 | 0.281 | 0.009 | No | Yes |
| CA020510138 | 0.285 | 0 | No | Yes |
| CA020510139 | 0.297 | 0.041 | No | Yes |
| CA020510140 | 0.287 | 0.011 | No | Yes |
| CA020510141 | 0.307 | 0.065 | No | Yes |
| CA020510143 | 0.304 | 0.06 | No | Yes |
| CA020510144 | 0.303 | 0.06 | No | Yes |
| CA020510145 | 0.285 | 0 | No | No |
| CA020510146 | 0.287 | 0 | No | No |
| CA020510147 | 0.293 | 0.0222 | No | Yes |
| CA020510148 | 0.299 | 0.04 | No | Yes |
| CA020610151 | 0.287 | 0 | No | No |
| CA020610152 | 0.288 | 0.01 | No | No |
| CA020610153 | 0.28 | 0 | No | Yes |
| CA020610155 | 0.323 | 0.109 | No | No |
| CA020610156 | 0.32 | 0.1311 | No | Yes |
| CA020610157 | 0.292 | 0 | No | Yes |
| CA020610158 | 0.285 | 0.018 | Yes | Yes |
| CA020610159 | 0.28 | 0.002 | No | Yes |
| CA020610162 | 0.215 | 0 | No | No |
| CA020610163 | 0.298 | 0.024 | No | No |
| CA020610164 | 0.289 | 0 | No | Yes |
| CA020610165 | 0.287 | 0 | No | Yes |
| CA020610166 | 0.283 | 0 | No | Yes |
| CA020610167 | 0.289 | 0.006 | No | No |
| CA020610168 | 0.284 | 0.014 | No | Yes |
| CA020610169 | 0.285 | 0 | No | Yes |
| CA020610170 | 0.287 | 0.007 | No | No |
| CA020610171 | 0.3 | 0.0342 | No | Yes |
| CA020610172 | 0.285 | 0.002 | Yes | Yes |
| CA020610173 | 0.284 | 0.008 | No | Yes |
| CA020610174 | 0.285 | 0.003 | No | No |
| CA020610175 | 0.277 | 0 | No | No |
| CA020610176 | 0.284 | 0 | No | Yes |
| CA020610177 | 0.277 | 0 | No | No |
| CA020610178 | 0.283 | 0.003 | No | Yes |
| CA020610179 | 0.285 | 0.009 | No | Yes |
| CA030710181 | 0.287 | 0 | No | No |
| CA030710182 | 0.298 | 0.026 | No | No |
| CA030710183 | 0.284 | 0 | No | No |
| CA030710184 | 0.28 | 0 | No | Yes |
| CA030710185 | 0.284 | 0 | No | Yes |
| CA030710186 | 0.294 | 0.023 | No | No |
| CA030710187 | 0.281 | 0 | Yes | Yes |
| CA030710188 | 0.352 | 0.227 | No | Yes |
| CA030710189 | 0.288 | 0.009 | No | No |
| CA030710190 | 0.283 | 0 | No | Yes |
| CA030710191 | 0.285 | 0.01 | No | No |
| CA030710192 | 0.291 | 0.001 | No | No |
| CA030710193 | 0.292 | 0.024 | No | Yes |
| CA030710195 | 0.286 | 0.001 | No | No |
| CA030710196 | 0.279 | 0 | No | No |
| CA030710197 | 0.281 | 0 | No | Yes |
| CA030710198 | 0.285 | 0.002 | No | No |
| CA030710200 | 0.258 | 0.001 | Yes | Yes |
| CA030710201 | 0.271 | 0 | Yes | Yes |
| CA030710202 | 0.286 | 0 | No | Yes |
| CA030710203 | 0.28 | 0 | Yes | Yes |
| CA030710204 | 0.283 | 0 | No | Yes |
| CA030710205 | 0.285 | 0 | No | No |
| CA030710206 | 0.286 | 0 | No | No |
| CA030710207 | 0.282 | 0 | No | No |
| CA030710208 | 0.281 | 0.002 | No | No |
| CA030810211 | 0.288 | 0 | Yes | Yes |
| CA030810212 | 0.288 | 0 | No | Yes |
| CA030810213 | 0.208 | 0.002 | No | Yes |
| CA030810214 | 0.287 | 0 | No | Yes |
| CA030810215 | 0.286 | 0 | No | No |
| CA030810216 | 0.288 | 0 | No | No |
| CA030810217 | 0.276 | 0 | Yes | No |
| CA030810218 | 0.262 | 0 | No | Yes |
| CA030810219 | 0.327 | 0.23 | No | Yes |
| CA030810220 | 0.302 | 0.072 | No | Yes |
| CA030810221 | 0.285 | 0 | No | No |
| CA030810222 | 0.28 | 0 | No | No |
| CA030810223 | 0.279 | 0 | No | No |
| CA030810224 | 0.303 | 0.066 | No | Yes |
| CA030810225 | 0.296 | 0.0306 | No | Yes |
| CA030810226 | 0.34 | 0.1827 | No | Yes |
| CA030810227 | 0.286 | 0 | No | No |
| CA030810228 | 0.28 | 0 | No | Yes |
| CA030810229 | 0.352 | 0.219 | No | Yes |
| CA030810230 | 0.279 | 0 | No | Yes |
| CA030810231 | 0.285 | 0 | No | No |
| CA030810232 | 0.282 | 0 | No | Yes |
| CA030810233 | 0.283 | 0 | No | Yes |
| CA030810235 | 0.283 | 0 | No | Yes |
| CA030810236 | 0.288 | 0.001 | No | Yes |
| CA030810237 | 0.284 | 0.004 | No | Yes |
| CA030810238 | 0.286 | 0.009 | No | Yes |
| CA030910241 | 0.225 | 0.008 | Yes | Yes |
| CA030910242 | 0.291 | 0 | No | Yes |
| CA030910243 | 0.276 | 0 | No | No |
| CA030910244 | 0.286 | 0 | No | No |
| CA030910245 | 0.295 | 0.027 | No | Yes |
| CA030910246 | 0.277 | 0 | No | Yes |
| CA030910247 | 0.344 | 0.177 | Yes | No |
| CA030910248 | 0.23 | 0.012 | No | Yes |
| CA030910249 | 0.29 | 0 | No | Yes |
| CA030910250 | 0.167 | 0.004 | No | Yes |
| CA030910251 | 0.289 | 0.008 | No | Yes |
| CA030910252 | 0.282 | 0 | No | No |
| CA030910253 | 0.301 | 0.0462 | No | Yes |
| CA030910254 | 0.284 | 0.001 | Yes | Yes |
| CA030910255 | 0.324 | 0.135 | No | Yes |
| CA030910256 | 0.288 | 0.005 | Yes | No |
| CA030910257 | 0.289 | 0.0346 | No | No |
| CA030910258 | 0.288 | 0 | No | No |
| CA030910259 | 0.29 | 0 | Yes | No |
| CA030910260 | 0.27 | 0 | No | No |
| CA030910261 | 0.285 | 0 | No | No |
| CA030910262 | 0.281 | 0.004 | No | Yes |
| CA030910263 | 0.297 | 0.029 | No | No |
| CA030910264 | 0.306 | 0.059 | No | Yes |
| CA030910265 | 0.287 | 0 | No | Yes |
| CA030910266 | 0.291 | 0.021 | No | Yes |
| CA030910267 | 0.288 | 0 | No | No |
| CA030910268 | 0.285 | 0 | No | Yes |
| CA031010271 | 0.285 | 0.003 | No | No |
| CA031010272 | 0.317 | 0.1198 | No | Yes |
| CA031010273 | 0.282 | 0.0016 | No | No |
| CA031010274 | 0.284 | 0 | No | No |
| CA031010275 | 0.302 | 0.05 | No | No |
| CA031010276 | 0.286 | 0 | No | No |
| CA031010277 | 0.285 | 0 | No | No |
| CA031010278 | 0.289 | 0.004 | No | Yes |
| CA031010279 | 0.291 | 0.0218 | No | Yes |
| CA031010280 | 0.283 | 0 | No | Yes |
| CA031010281 | 0.29 | 0.013 | Yes | No |
| CA031010282 | 0.283 | 0 | No | Yes |
| CA031010283 | 0.302 | 0.061 | No | Yes |
| CA031010284 | 0.285 | 0 | No | No |
| CA031010285 | 0.283 | 0 | No | Yes |
| CA031010286 | 0.285 | 0 | No | No |
| CA031010287 | 0.347 | 0.1996 | No | Yes |
| CA031010288 | 0.282 | 0 | No | No |
| CA031010289 | 0.288 | 0.002 | No | No |
| CA031010290 | 0.289 | 0 | No | No |
| CA031010291 | 0.283 | 0 | No | Yes |
| CA031010292 | 0.283 | 0 | No | Yes |
| CA031010293 | 0.285 | 0.0034 | No | No |
| CA031010294 | 0.262 | 0 | No | No |
| CA031010295 | 0.298 | 0.0568 | No | Yes |
| CA031010296 | 0.283 | 0 | No | Yes |
| CA031010298 | 0.331 | 0.165 | No | Yes |
| CA031010299 | 0.286 | 0 | No | Yes |
| CA031110301 | 0.283 | 0 | No | Yes |
| CA031110302 | 0.349 | 0.2056 | No | Yes |
| CA031110303 | 0.326 | 0.139 | No | Yes |
| CA031110304 | 0.277 | 0 | No | No |
| CA031110305 | 0.285 | 0.0028 | No | Yes |
| CA031110306 | 0.283 | 0 | No | No |
| CA031110307 | 0.286 | 0 | No | Yes |
| CA031110308 | 0.395 | 0.3441 | Yes | No |
| CA031110309 | 0.281 | 0 | No | No |
| CA031110310 | 0.285 | 0 | No | No |
| CA031110311 | 0.281 | 0 | No | Yes |
| CA031110312 | 0.278 | 0 | No | No |
| CA031110313 | 0.286 | 0.001 | No | No |
| CA031110314 | 0.275 | 0 | Yes | Yes |
| CA031110315 | 0.288 | 0.004 | No | No |
| CA031110316 | 0.279 | 0 | No | No |
| CA031110317 | 0.279 | 0.001 | No | No |
| CA031110318 | 0.287 | 0 | No | Yes |
| CA031110319 | 0.296 | 0.031 | No | No |
| CA031110320 | 0.284 | 0 | No | No |
| CA031110321 | 0.286 | 0.019 | No | Yes |
| CA031110322 | 0.286 | 0 | No | Yes |
| CA031110323 | 0.323 | 0.182 | No | Yes |
| CA031110324 | 0.28 | 0.008 | No | Yes |
| CA031110327 | 0.279 | 0 | No | Yes |
| CA031110328 | 0.279 | 0.004 | No | Yes |
| CA031210331 | 0.339 | 0.1691 | No | Yes |
| CA031210333 | 0.29 | 0 | No | No |
| CA031210335 | 0.286 | 0.006 | Yes | Yes |
| CA031210336 | 0.277 | 0 | Yes | No |
| CA031210337 | 0.273 | 0 | Yes | No |
| CA031210339 | 0.281 | 0 | No | No |
| CA031210340 | 0.397 | 0.3611 | No | No |
| CA031210342 | 0.277 | 0 | No | Yes |
| CA031210343 | 0.284 | 0 | No | No |
| CA031210344 | 0.289 | 0 | Yes | No |
| CA031210345 | 0.283 | 0 | No | No |
| CA031210346 | 0.283 | 0 | No | Yes |
| CA031210347 | 0.283 | 0.015 | No | Yes |
| CA031210348 | 0.286 | 0 | No | No |
| CA031210349 | 0.283 | 0 | No | No |
| CA031210350 | 0.302 | 0.0406 | Yes | No |
| CA031210351 | 0.251 | 0.005 | No | Yes |
| CA031210353 | 0.291 | 0.011 | No | No |
| CA031210354 | 0.282 | 0 | No | No |
| CA031210355 | 0.289 | 0 | No | No |
| CA031310361 | 0.289 | 0.005 | No | No |
| CA031310362 | 0.28 | 0 | No | No |
| CA031310363 | 0.283 | 0.004 | No | No |
| CA031310364 | 0.283 | 0 | No | Yes |
| CA031310365 | 0.286 | 0 | Yes | Yes |
| CA031310366 | 0.278 | 0 | No | No |
| CA031310367 | 0.285 | 0 | Yes | Yes |
| CA031310369 | 0.281 | 0 | No | Yes |
| CA031310371 | 0.279 | 0 | No | Yes |
| CA031310372 | 0.283 | 0 | No | Yes |
| CA031310373 | 0.287 | 0 | No | Yes |
| CA031310375 | 0.285 | 0 | Yes | Yes |
| CA031310376 | 0.299 | 0.037 | No | No |
| CA031310377 | 0.261 | 0.005 | No | Yes |
| CA031310378 | 0.202 | 0 | No | Yes |
| CA031310379 | 0.286 | 0.001 | No | Yes |
| CA031310380 | 0.282 | 0 | No | No |
| CA031310381 | 0.284 | 0 | Yes | Yes |
| CA031310382 | 0.277 | 0 | No | No |
| CA031310383 | 0.29 | 0 | No | Yes |
| CA031310384 | 0.284 | 0 | No | Yes |
| CA031310385 | 0.278 | 0 | No | Yes |
| CA031310386 | 0.282 | 0.005 | No | Yes |
| CA031310388 | 0.287 | 0.009 | No | Yes |
| CA031410391 | 0.282 | 0 | No | No |
| CA031410392 | 0.288 | 0 | No | No |
| CA031410393 | 0.283 | 0 | No | No |
| CA031410394 | 0.285 | 0 | No | No |
| CA031410396 | 0.261 | 0 | No | Yes |
| CA031410397 | 0.29 | 0 | No | Yes |
| CA031410398 | 0.28 | 0 | No | No |
| CA031410399 | 0.277 | 0 | Yes | Yes |
| CA031410400 | 0.282 | 0 | No | No |
| CA031410401 | 0.283 | 0.003 | No | No |
| CA031410402 | 0.287 | 0 | No | No |
| CA031410403 | 0.279 | 0.004 | No | Yes |
| CA031410404 | 0.287 | 0 | No | Yes |
| CA031410405 | 0.289 | 0 | No | Yes |
| CA031410406 | 0.291 | 0.03 | No | Yes |
| CA031410407 | 0.29 | 0 | No | No |
| CA031410408 | 0.284 | 0 | No | No |
| CA031410409 | 0.286 | 0 | No | No |
| CA031410410 | 0.286 | 0.01 | No | Yes |
| CA031410411 | 0.299 | 0.0332 | No | Yes |
| CA031410412 | 0.284 | 0 | No | No |
| CA031410413 | 0.285 | 0 | No | No |
| CA031410415 | 0.285 | 0 | No | No |
| CA031410416 | 0.285 | 0 | No | No |
| CA031410417 | 0.286 | 0 | No | No |
| CA041510421 | 0.288 | 0 | No | Yes |
| CA041510423 | 0.282 | 0 | Yes | Yes |
| CA041510424 | 0.289 | 0.01 | No | No |
| CA041510425 | 0.282 | 0 | No | No |
| CA041510426 | 0.286 | 0 | No | Yes |
| CA041510427 | 0.291 | 0 | No | Yes |
| CA041510428 | 0.287 | 0 | No | Yes |
| CA041510429 | 0.287 | 0 | No | Yes |
| CA041510430 | 0.285 | 0 | No | No |
| CA041510431 | 0.294 | 0.028 | No | Yes |
| CA041510432 | 0.289 | 0.009 | No | Yes |
| CA041510433 | 0.286 | 0.007 | No | Yes |
| CA041510434 | 0.282 | 0 | No | No |
| CA041510435 | 0.281 | 0 | Yes | Yes |
| CA041510436 | 0.303 | 0.031 | No | Yes |
| CA041510437 | 0.283 | 0 | No | Yes |
| CA041510438 | 0.279 | 0 | No | No |
| CA041510439 | 0.313 | 0.089 | No | Yes |
| CA041510440 | 0.284 | 0 | No | No |
| CA041510441 | 0.271 | 0 | No | Yes |
| CA041510442 | 0.285 | 0.0048 | No | Yes |
| CA041510443 | 0.283 | 0.001 | No | Yes |
| CA041510444 | 0.29 | 0.0044 | No | No |
| CA041510445 | 0.278 | 0 | No | No |
| CA041510446 | 0.278 | 0 | No | Yes |
| CA041510447 | 0.287 | 0 | No | Yes |
| CA041610451 | 0.29 | 0 | No | Yes |
| CA041610452 | 0.284 | 0.001 | No | Yes |
| CA041610453 | 0.288 | 0 | No | No |
| CA041610454 | 0.279 | 0 | No | Yes |
| CA041610455 | 0.282 | 0 | No | No |
| CA041610456 | 0.286 | 0 | No | No |
| CA041610457 | 0.292 | 0 | No | Yes |
| CA041610458 | 0.283 | 0 | No | Yes |
| CA041610460 | 0.288 | 0 | No | Yes |
| CA041610461 | 0.287 | 0 | No | No |
| CA041610462 | 0.281 | 0 | No | No |
| CA041610464 | 0.29 | 0 | Yes | No |
| CA041610465 | 0.28 | 0 | Yes | No |
| CA041610466 | 0.283 | 0 | No | Yes |
| CA041610467 | 0.285 | 0.003 | No | No |
| CA041610468 | 0.283 | 0 | Yes | Yes |
| CA041610469 | 0.275 | 0 | No | Yes |
| CA041610470 | 0.24 | 0 | Yes | No |
| CA041610471 | 0.286 | 0 | No | No |
| CA041610472 | 0.28 | 0 | No | No |
| CA041610473 | 0.283 | 0 | No | Yes |
| CA041610475 | 0.284 | 0 | No | Yes |
| CA041610476 | 0.29 | 0 | No | Yes |
| CA041610477 | 0.288 | 0 | Yes | No |
| CA041610478 | 0.265 | 0 | Yes | No |
| CA041710481 | 0.284 | 0 | No | Yes |
| CA041710482 | 0.288 | 0 | No | No |
| CA041710483 | 0.287 | 0 | No | No |
| CA041710484 | 0.278 | 0 | No | No |
| CA041710485 | 0.28 | 0 | No | No |
| CA041710486 | 0.286 | 0 | No | Yes |
| CA041710487 | 0.281 | 0 | Yes | No |
| CA041710488 | 0.21 | 0 | Yes | Yes |
| CA041710489 | 0.282 | 0 | No | No |
| CA041710490 | 0.29 | 0 | No | No |
| CA041710491 | 0.298 | 0.032 | No | No |
| CA041710492 | 0.285 | 0.004 | No | Yes |
| CA041710493 | 0.286 | 0 | No | No |
| CA041710494 | 0.288 | 0 | No | Yes |
| CA041710495 | 0.285 | 0 | No | No |
| CA041710496 | 0.283 | 0 | No | No |
| CA041710497 | 0.284 | 0 | No | No |
| CA041710498 | 0.282 | 2.00E-04 | No | No |
| CA041710499 | 0.285 | 0 | No | No |
| CA041710500 | 0.284 | 0 | No | No |
| CA041710501 | 0.282 | 0 | Yes | No |
| CA041710502 | 0.286 | 0 | No | No |
| CA041710503 | 0.285 | 0 | No | No |
| CA041710504 | 0.284 | 0 | No | No |
| CA041710505 | 0.286 | 0 | No | No |
| CA041710506 | 0.218 | 0 | No | Yes |
| CA041710507 | 0.287 | 0 | Yes | No |
| CA051810511 | 0.28 | 0 | No | No |
| CA051810512 | 0.279 | 0 | No | No |
| CA051810513 | 0.287 | 0 | No | No |
| CA051810514 | 0.287 | 0.007 | No | Yes |
| CA051810515 | 0.286 | 6.00E-04 | No | No |
| CA051810516 | 0.286 | 0 | No | No |
| CA051810518 | 0.288 | 0 | No | Yes |
| CA051810519 | 0.282 | 0 | No | No |
| CA051810520 | 0.283 | 0 | No | No |
| CA051810521 | 0.293 | 0.033 | No | Yes |
| CA051810522 | 0.307 | 0.1159 | No | Yes |
| CA051810523 | 0.285 | 0 | No | Yes |
| CA051810524 | 0.287 | 0.014 | No | Yes |
| CA051810525 | 0.288 | 0.014 | No | No |
| CA051810526 | 0.289 | 0 | No | No |
| CA051810527 | 0.284 | 0 | No | Yes |
| CA051810528 | 0.286 | 0 | No | No |
| CA051810529 | 0.283 | 0 | No | No |
| CA051810530 | 0.284 | 0 | No | Yes |
| CA051810532 | 0.284 | 0 | No | No |
| CA051810533 | 0.284 | 0 | No | No |
| CA051810534 | 0.283 | 0.004 | No | No |
| CA051810535 | 0.285 | 0.0012 | Yes | Yes |
| CA051810536 | 0.284 | 0 | No | Yes |
| CA051810537 | 0.288 | 0.002 | No | No |
| CA051810538 | 0.282 | 0 | No | Yes |
| CA051910541 | 0.185 | 0 | Yes | Yes |
| CA051910542 | 0.291 | 0 | No | Yes |
| CA051910543 | 0.286 | 0.02 | No | Yes |
| CA051910544 | 0.283 | 0 | No | Yes |
| CA051910545 | 0.285 | 2.00E-04 | No | Yes |
| CA051910546 | 0.284 | 0 | No | Yes |
| CA051910547 | 0.288 | 0.008 | No | No |
| CA051910548 | 0.287 | 0 | No | Yes |
| CA051910549 | 0.287 | 0 | No | No |
| CA051910550 | 0.284 | 0 | No | No |
| CA051910551 | 0.266 | 0 | Yes | No |
| CA051910554 | 0.214 | 0 | No | Yes |
| CA051910555 | 0.281 | 0 | No | No |
| CA051910556 | 0.286 | 0 | Yes | No |
| CA051910557 | 0.287 | 0.004 | Yes | Yes |
| CA051910558 | 0.245 | 0 | Yes | No |
| CA051910559 | 0.29 | 0.022 | No | Yes |
| CA051910560 | 0.285 | 0 | Yes | Yes |
| CA051910561 | 0.286 | 2.00E-04 | No | No |
| CA051910562 | 0.287 | 0.001 | No | Yes |
| CA051910563 | 0.285 | 0 | No | Yes |
| CA051910564 | 0.288 | 0 | No | Yes |
| CA051910565 | 0.279 | 0 | No | Yes |
| CA051910566 | 0.285 | 0.015 | No | Yes |
| CA051910567 | 0.279 | 0 | No | No |
| CA051910569 | 0.286 | 0 | No | No |
| CA052010571 | 0.287 | 0 | Yes | No |
| CA052010572 | 0.28 | 0 | No | Yes |
| CA052010574 | 0.285 | 0 | No | Yes |
| CA052010575 | 0.281 | 0 | No | Yes |
| CA052010576 | 0.28 | 0 | Yes | No |
| CA052010577 | 0.287 | 0 | Yes | No |
| CA052010578 | 0.289 | 0 | Yes | Yes |
| CA052010579 | 0.285 | 0 | No | Yes |
| CA052010580 | 0.285 | 0.001 | Yes | No |
| CA052010581 | 0.283 | 0 | Yes | Yes |
| CA052010582 | 0.28 | 0 | No | No |
| CA052010583 | 0.277 | 0 | No | Yes |
| CA052010584 | 0.289 | 0.012 | No | Yes |
| CA052010585 | 0.285 | 0 | No | Yes |
| CA052010586 | 0.281 | 0 | Yes | Yes |
| CA052010587 | 0.238 | 0 | Yes | No |
| CA052010588 | 0.288 | 0 | No | Yes |
| CA052010589 | 0.283 | 0 | No | No |
| CA052010590 | 0.283 | 8.00E-04 | Yes | Yes |
| CA052010591 | 0.282 | 0.015 | No | No |
| CA052010592 | 0.283 | 0 | No | Yes |
| CA052010593 | 0.284 | 0 | No | Yes |
| CA052010594 | 0.287 | 0 | Yes | No |
| CA052010595 | 0.289 | 0.007 | No | No |
| CA052010596 | 0.283 | 0 | No | No |
| CA052010597 | 0.286 | 0 | No | No |
